# *Inocybe brijunica* sp. nov., a New Ectomycorrhizal Fungus from Mediterranean Croatia Revealed by Morphology and Multilocus Phylogenetic Analysis

**DOI:** 10.3390/jof7030199

**Published:** 2021-03-10

**Authors:** Armin Mešić, Danny Haelewaters, Zdenko Tkalčec, Jingyu Liu, Ivana Kušan, M. Catherine Aime, Ana Pošta

**Affiliations:** 1 Laboratory for Biological Diversity, Ruđer Bošković Institute, Bijenička cesta 54, HR-10000 Zagreb, Croatia; amesic@irb.hr (A.M.); ztkalcec@irb.hr (Z.T.); ikusan@irb.hr (I.K.); aposta@irb.hr (A.P.); 2 Faculty of Science, University of South Bohemia, Branišovská 31, 370 05 České Budějovice, Czech Republic; 3 Department of Botany and Plant Pathology, Purdue University, 915 W. State Street, West Lafayette, IN 47907, USA; liu1643@purdue.edu (J.L.); maime@purdue.edu (M.C.A.); 4 Research Group Mycology, Department of Biology, Ghent University, K.L. Ledeganckstraat 35, 9000 Ghent, Belgium

**Keywords:** 1 new taxon, Agaricomycetes, Basidiomycota, biodiversity, climate change, Inocybaceae, taxonomy

## Abstract

A new ectomycorrhizal species was discovered during the first survey of fungal diversity at Brijuni National Park (Croatia), which consists of 14 islands and islets. The National Park is located in the Mediterranean Biogeographical Region, a prominent climate change hot-spot. *Inocybe brijunica* sp. nov., from sect. *Hysterices* (Agaricales, Inocybaceae), is described based on morphology and multilocus phylogenetic data. The holotype collection was found at the edge between grassland and *Quercus ilex* forest with a few planted *Pinus pinea* trees, on Veli Brijun Island, the largest island of the archipelago. It is easily recognized by a conspicuous orange to orange–red–brown membranaceous surface layer located at or just above the basal part of the stipe. Other distinctive features of *I. brijunica* are the medium brown, radially fibrillose to rimose pileus; pale to medium brown stipe with fugacious cortina; relatively small, amygdaliform to phaseoliform, and smooth basidiospores, measuring ca. 6.5–9 × 4–5.5 µm; thick-walled, utriform, lageniform or fusiform pleurocystidia (lamprocystidia) with crystals and mostly not yellowing in alkaline solutions; cheilocystidia of two types (lamprocystidia and leptocystidia); and the presence of abundant caulocystidia only in the upper 2–3 mm of the stipe. Phylogenetic reconstruction of a concatenated dataset of the internal transcribed spacer region (ITS), the nuclear 28S rRNA gene (nrLSU), and the second largest subunit of RNA polymerase II (*rpb2*) resolved *I. brijunica* and *I. glabripes* as sister species.

## 1. Introduction

The Brijuni archipelago consists of 14 islands and islets located in the Adriatic Sea (northern Mediterranean, Europe), near the southwestern coast of the Istrian peninsula. The archipelago is home to Brijuni National Park [1], which covers 33.9 km^2^ of protected area, including the surrounding sea. The islands’ surface area covers 7.4 km^2^; Veli Brijun is the largest island with 5.7 km^2^ and is devoid of permanent inhabitants. The National Park was established in 1983 to protect valuable marine and coastal (land) ecosystems and their biodiversity. The area is floristically rich, covered with evergreen vegetation and home to more than 400 native and exotic plant species mostly of Mediterranean origin. The Brijuni archipelago is characterized by a northern Mediterranean climate [1] with an average annual temperature of 13.9 °C, annual average precipitation of 817 mm, and a relatively high average air humidity of 76%.

During the 20th century, the air temperature increased globally by 0.74 °C, but the temperature rise in the Mediterranean area was higher—up to 1.5–4 °C depending on the region [2]. Following the Regional Climate Change Index (RCCI), the Mediterranean region is one of the most prominent climate change hot-spots in the world [3,4]. Models predicting the intensity of future climate change in this area are not optimistic. According to Mariotti et al. [5], land areas of the Mediterranean will gradually become drier; models predict 8% less precipitation in 2020–2049 compared to 1950–2000, a number that is projected to increase to 15% in 2070–2099. Drying in the northern Mediterranean [4,6] is projected to occur year-round, which will increase water stress for ecosystems if climate change continues at the current rate. Therefore, in the future, we can expect an increase of devastating climatic events (floods, storms, and droughts), more attacks of organisms that cause diseases, and a higher number of invasive species that will compete with indigenous species populations. These events could have a strong negative impact on the Mediterranean forest ecosystems [2] as well as on fruiting and existence of drought-sensitive fungal species in the area [7].

The ratio of plant species to macrofungal species is conservatively estimated as 1:6 [8]. Given the high number of plant species in the Brijuni Archipelago, an equally high diversity of fungal species is expected. Currently, however, there are no published data on fungi from this area. And even though fungal taxonomy has a long history in Europe, many species continue to be described from the continent. In 2019, 23% of newly described species of fungi were from Europe [9]. Given all this, it can be expected that undescribed species may be discovered at the Brijuni Islands. Initial field trips by Croatian mycologists aiming to document the fungal diversity of Brijuni National Park were carried out during the fall season in 2014, 2015, 2016, and 2020. In total, 546 records of basidiomycete fungi were made; 184 samples were collected and deposited in the Croatian National Fungarium (CNF) in Zagreb, Croatia. One of the most common genera found was *Inocybe* (Fr.) Fr. (Agaricomycetes, Agaricales, Inocybaceae), with 28 collections.

*Inocybe sensu lato* (s.l.) is a highly diverse genus of ectomycorrhizal mushrooms [10] currently with about 1000 accepted species [11]. It belongs to the family Inocybaceae Jülich. The species diversity within *Inocybe* s.l. is best known in Europe, especially in its central countries—Austria, France, Germany, the Netherlands, and Switzerland—with more than 450 species recorded [12]. Current ongoing studies are exploring the diversity of the genus in Europe and many new species have been recently described [13,14,15,16,17,18,19,20,21,22]. 

According to the taxonomic treatment by Matheny et al. [23] based on a six-locus phylogeny, the family Inocybaceae now consists of seven genera: *Auritella* Matheny & Bougher, *Inocybe sensu stricto* (s.s.), *Inosperma* (Kühner) Matheny & Esteve-Rav., *Mallocybe* (Kuyper) Matheny, Vizzini & Esteve-Rav., *Nothocybe* Matheny & K.P.D. Latha, *Pseudosperma* Matheny & Esteve-Rav., and *Tubariomyces* Esteve-Rav. & Matheny. The largest genus remains *Inocybe* s.s. with about 850 known species worldwide. Members of *Inocybe* s.s. can be distinguished from other genera in the family by the presence of pleurocystidia and basidiospores (with a distinct apiculus) that range from amygdaliform to ellipsoid, subcylindrical, angular, nodulose, or spinose in shape [23].

On 16 November 2016, during our fungal diversity research on the island of Veli Brijun, basidiomata of an interesting fungus belonging to *Inocybe* s.s. were found. Its basidiomata were macroscopically striking by the presence of an orange to orange–red–brown membranaceous surface layer (possibly a remnant of universal veil) in the basal part of the stipe—an unusual feature in the genus. Further detailed molecular phylogenetic and morphological analyses confirmed that the species was hitherto unknown to science. Therefore, it is here described as *I. brijunica* sp. nov.

## 2. Materials and Methods

### 2.1. Description of the Research Area

The holotype collection of *Inocybe brijunica* was collected on Veli Brijun Island. The biogeographical position and a long history of human interventions have shaped the landscape of Veli Brijun Island, merging natural and anthropogenic elements. The island is mostly covered by a thermophilous forest with holm oak (*Quercus ilex*) (including those in the maquis degradation stage), planted alleys or groves of pine trees (*Pinus halepensis* Mill. and *P. pinea* L.), cypresses (*Cupressus sempervirens* L.), cedars (*Cedrus* spp.), and parks and lawns often used as golf courses. 

The *Inocybe* collection was found on the edge of the mature thermophilous *Q. ilex* forest and a lawn grazed by large herbivores (fallow deer [*Dama dama* L.], axis deer [*Axis axis* Erxleben], and European mouflon [*Ovis gmelini musimon* Pall.]) and occasionally machine-mowed by park staff. In addition, a few mature planted trees of *P. pinea* were present at the forest edge. Basidiomata of *I. brijunica* were found at ca. 70 m from the sea, epigeous on the soil covered with a shallow layer of oak and pine litter intermixed with scattered short grasses. The understory of the surrounding forest was devoid of herbaceous plants and shrubs due to the presence of large herbivores.

### 2.2. Morphological Study

The species description is based on a single but large collection consisting of 20 basidiomata. Macroscopic characters were documented with a Canon EOS 5D digital camera equipped with a Canon MR-14EX macro ring flash (Canon Europe, Uxbridge, UK). Microscopic features were observed by brightfield and phase contrast microscopy using a BX51 optical microscope (Olympus, Hamburg, Germany) under magnification up to 1500× and photographed with a Canon EOS M50 digital camera. Descriptions and images of microscopic characters were made from rehydrated specimens mounted in 2.5% potassium hydroxide (KOH), except for cystidia that were observed in 3% ammonium hydroxide (NH_4_OH). Micromorphological terminology mostly follows Clémençon [24]. Line drawings were made by J.L. with PITT artist pens (Faber–Castell, Nürnberg, Germany) based on digital images.

Amyloid and dextrinoid reactions of basidiospores were tested in Melzer’s reagent [25]. Randomly selected basidiospores from photographs of lamellae mounts were measured with Motic Images Plus 2.0 software (Motic Europe, Barcelona, Spain). The length/width ratio of basidiospores is given as the “Q” value (min–av.–max). Average basidiospore and pleurocystidia lengths, widths, and Q values are shown in italics. Numbers in square brackets [X/Y/Z] denote X elements measured in Y basidiomata of Z collections. Measurements of cystidia do not include crystals present at the apex. Type material was preserved by drying on a flow of hot air at maximum temperature of 50 °C. The holotype is deposited at CNF, and an isotype is deposited at PUL (Kriebel Herbarium, Purdue University, West Lafayette, IN, USA).

### 2.3. DNA Extraction, PCR Amplification, and Sequencing

Genomic DNA was extracted from parts of the lamellae using the QIAamp DNA Micro Kit (Qiagen, Valencia, CA, USA). The first ~1100 bp of the nuclear 18S nuclear ribosomal RNA gene (nrSSU), the internal transcribed spacer region of the rDNA (ITS, consisting of ITS1–5.8S–ITS2), the first ~1400 bp of the nuclear 28S rRNA gene (nrLSU), and the second largest subunit of RNA polymerase II gene (*rpb2*) were amplified [26]. The following primers were used: NS1, NS2, and NS4 [27] for nrSSU; ITS9mun [28] and ITS4 [27] for ITS; LR0R, LR5, and LR7 for nrLSU [29,30]; and RBP2-b6F, RPB2-b7R, and RPB2-b7.1R for *rpb2* [31]. Amplifications were done in 25 μL reactions, containing 12.5 μL of Promega 2× PCR Master Mix (Promega Co., Madison, WI, USA), 1.25 μL of each 10 μM primer, 9.0 μL of H_2_O, and 1.0 μL of template DNA. PCR conditions for nrSSU, ITS, and nrLSU followed Haelewaters et al. [32]. For *rpb2*, PCR conditions were as follows: initial denaturation at 95 °C for 5:00 min; followed by 40 cycles of denaturation at 95 °C for 30 s, annealing at 55 °C for 45 s, and extension at 72 °C for 45 s; and final extension at 72 °C for 7:00 min. All amplifications were performed using the Eppendorf Mastercycler EP Thermal Cycler (Hauppauge, NY, USA). Purification of successful PCR products and sequencing in both directions using the amplification primers were outsourced to Genewiz (South Plainfield, NJ, USA). Sequence reads were assembled and edited using Sequencher 5.4.6 for Windows software (Gene Codes Corporation, Ann Arbor, MI, USA). Assembled sequences were deposited at the National Center for Biotechnology Information (NCBI) GenBank database, under accession numbers MN749503–MN749504 (nrSSU), MN749370–MN749371 (ITS), MN749492–MN749493 (nrLSU), and MT878448–MT878449 (*rpb2*).

### 2.4. Sequence Alignment and Phylogenetic Analysis

Newly obtained ITS sequences were BLAST searched against NCBI GenBank’s standard *nr*/*nt* nucleotide database (https://blast.ncbi.nlm.nih.gov/Blast.cgi, accessed on 9 August 2020), resulting in three isolates of *Inocybe glabripes* Ricken (sect. *Hysterices* Stangl & J. Veselský) as top results, which shared between 91.49% and 91.90% identity (GenBank accession numbers KX602255, MH216096, MN947389). Following this result, ITS, nrLSU, and *rpb2* sequences of *Inocybe* sect. *Hysterices* species [26,31,33,34,35] were downloaded for phylogenetic analysis.

Sequences were aligned by locus using MUSCLE version 3.7 [36], available through the Cipres Science Gateway [37]. Next, sequences in the ITS dataset were trimmed at the conserved motifs 5′–CATTA–3′ (3′ end of the nrSSU) and 5′–GACCT(CAAA…)–3′ (5′ end of the nrLSU) [38]. Because the different portions of the ITS spacer region (the two spacers and 5.8S) have different rates of evolution [39,40], the ITS1 and ITS2 spacers and the 5.8S conserved gene were extracted and treated as individual partitions in the multilocus analysis. Sequences in the nrLSU dataset were also trimmed to start with the conserved motif 5′–GACCT(CAAA…)–3′. Ambiguously aligned regions were removed using trimAl version 1.3 [41], with -gt = 0.6 and -cons = 0.5.

Evolutionary models for nucleotide substitution were selected for each partition (ITS1, 5.8S, ITS2, nrLSU, *rpb2*) using ModelFinder Plus [42], considering the Akaike Information Criterion. The data for each locus were combined in MEGA7 [43] to create a supermatrix of 2752 characters for 28 isolates representing ten species in *Inocybe* sect. *Hysterices* and two species in *Inocybe* sect. *Lactiferae* serving as outgroup taxa (details in Table 1). Maximum likelihood (ML) was inferred under partitioned models using IQ-TREE 1.6.7 [44,45]. Ultrafast bootstrapping was done with 1000 replicates [46].

## 3. Results

### 3.1. Phylogenetic Inference

**Figure 1:**
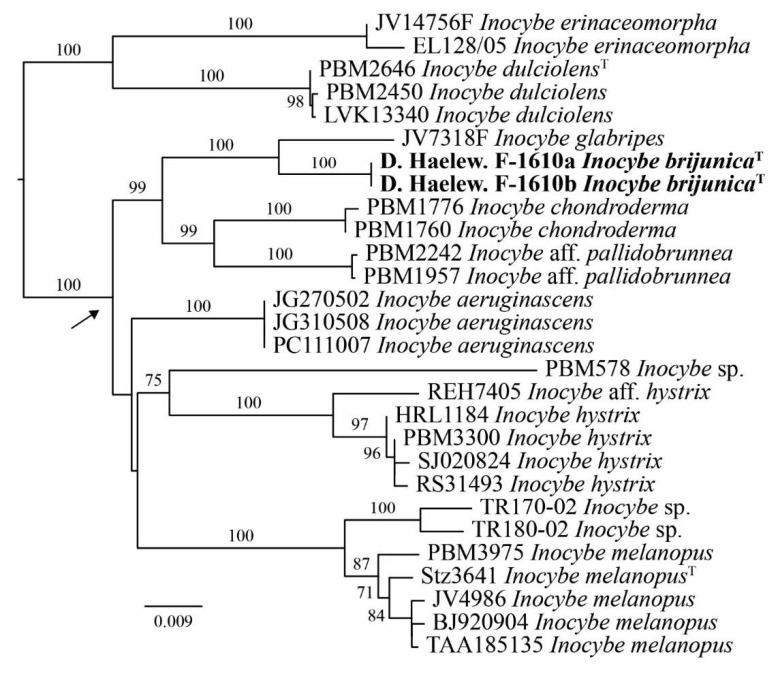
The best-scoring ML tree (-lnL = 8722.045120) of *Inocybe* sect. *Hysterices* (represented by the arrow) reconstructed from a concatenated ITS–nrLSU–*rpb2* dataset of 28 isolates. The tree topology is the result of ML inference performed in IQ-TREE. For each node, the ML bootstrap (≥70) is presented above or in front of the branch leading to that node. The new species is in boldface. ^T^ stands for type specimens.

The final multilocus dataset (Supplementary File S1) consists of 2752 characters, of which 425 are parsimony-informative and 2197 are constant. The number of total and parsimony-informative characters by locus as well as their selected evolutionary models as selected by ModelFinder Plus are presented in Table 2. The best-scoring ML tree (-lnL = 8722.045120) is shown in Figure 1. The topology is mostly congruent with Matheny and Kudzma [26], although support has improved for certain nodes. *Inocybe brijunica* sp. nov. is retrieved as a sister species of *I. glabripes* with maximum support. This set (*I. brijunica*, *I. glabripes*) is highly supported as sister to the clade holding *I. chondroderma* D.E. Stuntz ex Matheny, Norvell & E.C. Giles and *I.* aff. *pallidobrunnea* Kauffman.

### 3.2. Taxonomy

#### *Inocybe brijunica* Mešić, Tkalčec & Haelew., sp. nov.

**Figure 2:**
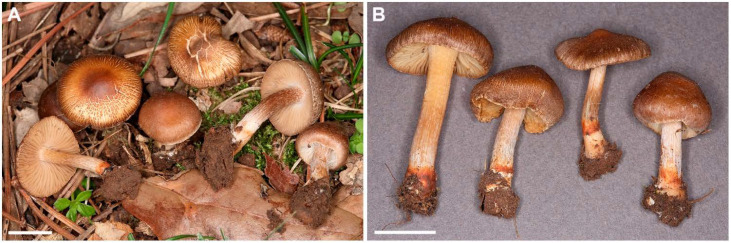
*Inocybe brijunica* (CNF 1/7345, holotype). (**A**) Basidiomata in situ. (**B**) Basidiomata in lab. Bars: A, B = 10 mm.

**Figure 3:**
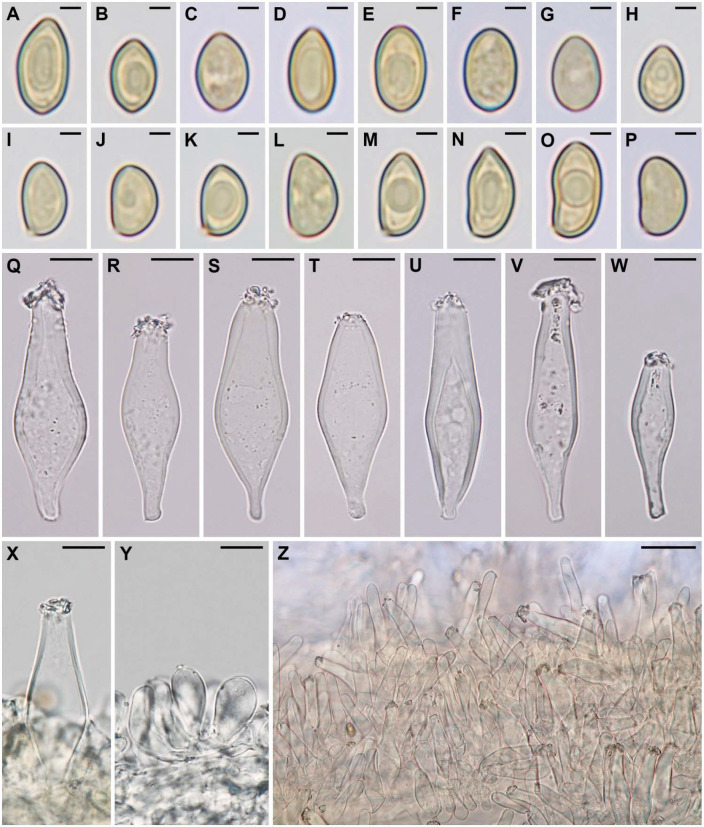
Inocybe brijunica (CNF 1/7345, holotype). (**A**–**H**) Basidiospores in frontal view. (**I**–**P**) Basidiospores in side view. (**Q**–**W**) Pleurocystidia. (**X**) Cheilolamprocystidium. (**Y**) Cheiloleptocystidia. (**Z**) Caulocystidia. Bars: (**A**–**P**) = 2 µm, (**Q**–**Y**) = 10 µm, Z = 30 µm.

**Figure 4:**
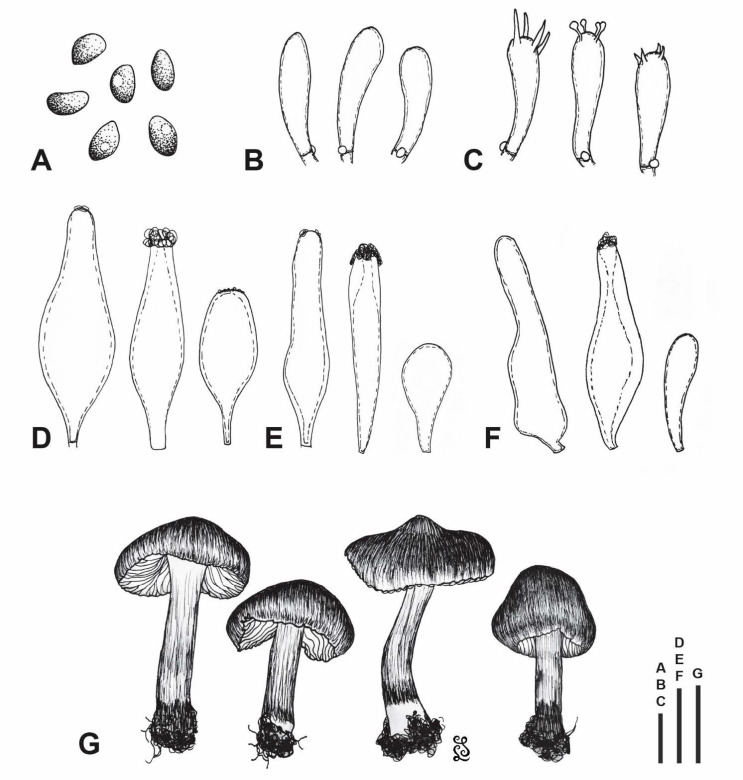
*Inocybe brijunica* (CNF 1/7345, holotype). (**A**) Basidiospores. (**B**) Basidioles. (**C**) Basidia. (**D**) Pleurocystidia. (**E**) Cheilocystidia). (**F**) Caulocystidia. (**G**) Basidiomata. Bars: (**A**–**C**) = 10 µm; (**D**–**F**) = 20 µm; (**G**) = 10 mm.

Figure 2, Figure 3 and Figure 4.

Mycobank MB838152

*Typification*: CROATIA. ISTRIA COUNTY: Brijuni National Park, Veli Brijun Island, 44°55′04″ N 13°46′33″ E, on the edge of grassland and forest of *Quercus ilex* L. with a few planted *Pinus pinea* L. trees along the forest edge, 16 November 2016, *A. Mešić & Z. Tkalčec* (holotype, CNF 1/7345; isotype, PUL F27673). GenBank (ex-isotype DNA isolate D. Haelew. F-1610a): nrSSU = MN749503, ITS = MN749370, nrLSU = MN749492, *rpb2* = MT878448; (ex-isotype DNA isolate D. Haelew. F-1610b): nrSSU = MN749504, ITS = MN749371, nrLSU = MN749493, *rpb2* = MT878449.

*Etymology*: Referring to the Brijuni archipelago, where the holotype was collected.

*Description:* Pileus 15–22 mm wide, obtusely (sub)conical with inflexed margin when young; convex to plano-convex, subumbonate and with deflexed margin at maturity; margin entire, occasionally with short radial splits; surface dry, finely radially fibrillose at first, then intensely fibrillose, rimulose to rimose, finally often partially cracked in small, shallow patches showing the paler flesh underneath; mostly medium brown, often with orange (fulvous brown) or reddish tones, less often light or dark brown where more deeply cracked; younger basidiomata often with rather inconspicuous, fibrillous, whitish veil remnants in marginal zone. Lamellae adnexed, subcrowded, L = ca. 40–50, l = 1–3, (sub)ventricose; whitish at first, then pale yellowish brown, finally light brown; edges fimbriate to slightly eroded, ± concolorous with sides. Stipe 17−30 × 2.5−4.5 mm, subcylindrical with slightly to moderately broadened base (up to 7 mm, sometimes submarginate); solid, surface dry, white flocculose at apex, becoming whitish longitudinally fibrillose toward base, fibrils more scattered with age, beneath the fibrils pale to medium brown; with more or less developed orange to dull orange–red or orange–red–brown, adhering, membranaceous surface layer (possibly a remnant of universal veil), at or just above the basal part of the stipe; basal tomentum scanty, whitish. Cortina (partial veil) present in young basidiomata, fibrillous, white, fugacious. Context cream, light brown when moist, not changing color on bruising, not darkening on drying. Odor spermatic. Taste not recorded.

Basidiospores [300/3/1] (6.2–)6.6–7.5–8.8(–9.8) × (4–)4.3–4.7–5.3(–5.7) µm, averages of different basidiomata 7.3–7.6 × 4.6–4.7 µm, Q = 1.35–1.6–1.96, av. Q = 1.58–1.62, a few very large spores occasionally present (up to ca. 12 × 7 µm); in frontal view ellipsoid, oblong or ovoid with rounded to subacute base and rounded to acute apex, in side view amygdaliform to phaseoliform, rarely subellipsoid, with rounded base and rounded to acute apex, sometimes subangulate in both views (especially in upper part); smooth, often with small, rather indistinct, apical germ-pore, moderately thick-walled (up to 0.8 µm), pale yellow–brown in KOH, pale brown in H_2_O, non-amyloid and non-dextrinoid. Basidia 20–30 × 6.5–9 µm, clavate, predominantly 4-spored, occasionally 2-spored, thin-walled, hyaline to yellowish. Pleurocystidia of lamprocystidia-type, very abundant, [90/4/1] 34–50–65(–70) × 9–14–21 µm, Q = 2.39–3.65–5.45, predominantly utriform, lageniform, or fusiform, with obtuse apex of 6–9(–11) µm wide, sometimes (sub)clavate, conical with obtuse apex, narrowly ellipsoid or subcylindrical, in alkaline solutions mostly (sub)hyaline, less often with slightly yellowish wall, sometimes with dirty yellow cytoplasmic pigment, with strongly to poorly developed crystals at apex (soluble in KOH, rarely lacking), thick-walled, wall most often gradually thickened towards the apex (up to 1–5.5 µm). Lamellar edge heterogeneous. Cheilocystidia of two types: (a) lamprocystidia similar to pleurocystidia (although more often without crystals), scattered to abundant, and (b) leptocystidia (paracystidia) 9–30 × 5–14 µm, mostly clavate, less often (sub)fusiform or utriform, hyaline to subhyaline, thin to moderately thick-walled (up to ca. 0.6 µm), scattered to abundant. Pileipellis a cutis, composed of repent, thin-walled, smooth to minutely encrusted, hyaline to pale yellow–brown hyphae, 1–5(–7) µm wide. Cells of the upper part of pileal context with brown, intracellular and partially also encrusted extracellular pigment (brown pigmented layer ca. 80–150 µm wide). Stipitipellis a cutis, composed of repent, thin-walled, smooth, ca. 2–10 µm wide hyphae. Caulocystidia mostly abundant (often crowded) in upper 2–3 mm of stipe length, sparsely present toward middle of the stipe; many in the form of lamprocystidia, quite similar to pleurocystidia, others very variable, narrowly utriform, lagenifom, (sub)cylindrical, clavate, urticoid or rather irregular, sometimes with subcapitate apex, some septate, hyaline, thin- to moderately thick-walled (up to ca. 1 µm); 12–100(–150) × 4–20 µm. Clamp connections present, conspicuous, rather abundant in all tissues.

*Distribution and ecology*: Thus far only known from the holotype collection. Ectomycorrhizal, found in the Mediterranean region of Croatia (Europe), on the island of Veli Brijun in Brijuni National Park, on the edge of *Quercus ilex* forest, with a few planted *Pinus pinea* trees, edging a neighboring grassland. An ITS sequence with accession number MH310748 [47], identified as *Inocybe* sp., from Italy shares 99% identity with *I. brijunica* (identities = 684/688 bp, gaps = 4/688) and may indicate a broader distribution in the Mediterranean basin.

## 4. Discussion

The results of our multilocus phylogenetic analysis and morphological study place *I. brijunica* in sect. *Hysterices* [48]. Basidiomata produced by species belonging to this section (in the original sense) possess a squamulose pileus and stipe, lack violaceous tones, and have amygdaliform basidiospores. Matheny and Kudzma [26] emended the section to include taxa with non-squarrose basidiomata. Macromorphologically, *I. brijunica* can be easily recognized from all other *Inocybe* species by a conspicuous orange to orange–red–brown membranaceous surface layer present at or just above the basal part of the stipe. Other important morphological characters are: medium brown pileus with radially fibrillose to rimose surface; pale to medium brown stipe with slightly to moderately broadened (or sometimes submarginate) base; presence of fugacious cortina in young basidiomata; spermatic odor; color of context unchanged upon bruising; relatively small, amygdaliform to phaseoliform (and sometimes subangulate), smooth basidiospores (ca. 6.5–9 × 4–5.5 µm); pleurocystidia as utriform, lageniform, or fusiform, thick-walled (up to 1–5.5 µm) lamprocystidia, mostly with crystals and not yellowing in alkaline solutions; cheilocystidia of two types (lamprocystidia and leptocystidia); and presence of abundant caulocystidia only in the upper 2–3 mm of stipe length.

The basidiospores of the morphologically and phylogenetically closest species, *I. glabripes*, are very similar in size, measuring ca. 6–8 × 4–5 µm [49,50], but they are readily distinguished by being amygdaliform but not phaseoliform as in *I. brijunica.* In addition, the cystidial walls of *I. glabripes* are thinner (up to 2(–2.5) µm thick). *Inocybe glabripes* is a widespread species occurring in parks and open woodlands on predominantly alkaline soils from the Mediterranean region to the boreal zone of Europe [51], which forms ectomycorrhizae exclusively with broadleaved trees. So far, the species has been found in symbiotic relationship with trees in the genera *Betula* L., *Fagus* L., *Populus* L., *Quercus* L., *Tilia* L. [49,50,51], and with *Castanea sativa* Mill. [52]. It can be expected that its sister species *I. brijunica* also forms ectomycorrhizal relationships only with broadleaved trees, such as *Quercus ilex* at the holotype locality.

*Inocybe pseudobrunnea* Alessio, which grows under *Abies alba* Mill. (Pinales, Pinaceae), has a similar phaseoliform, but somewhat larger basidiospores, 8.5–10.5(11) × 4.5–6 µm, and its cystidia are rather bright yellow in ammonia solution [53], a characteristic that rarely occurs in *I. brijunica*. *Inocybe gracilenta* E. Ludw., only known from the type collection in Sweden, a damp locality under *Alnus* sp. (Fagales, Betulaceae), *Populus tremula* L. and *Salix* sp. (Malpighiales, Salicaceae), has amygdaliform but not phaseoliform basidiospores, which are otherwise similar in size compared to *I. brijunica*, 7–8.5(9.5) × 4.5–5.5 µm [51]. Additional differences are the papillate pileus and the slenderer (up to 2 mm wide) and white to faintly cream-colored stipe [51]. The Mediterranean species *I. barrasae* Esteve-Rav., described from Spain and fruiting in spring (April–May) in thermophilous Mediterranean *Quercus*–*Cistus* forests, has amygdaliform-shaped basidiospores that are larger and more elongated (8–11.5 × 4.5–5.5 µm, av. Q = 1.85), and bright yellow-colored cystidia in ammonia solution [54]. *Inocybe aerea* E. Ludw., another species only known from the holotype collection in Germany [51], has amygdaliform to ovoid and slightly larger basidiospores (7.5–10.5 × 5–6 µm), an ochraceous yellow and more slender (up to 2 mm wide) stipe, and thinner-walled cystidia (walls 0.2–2 (–3) µm thick). The North American species *I. pyrotricha* Stuntz [55] has orange to cinnamon or rusty–red fibrils on the stipe, like *I. brijunica*, but differs by slightly larger (7.5–10 × 4.5–6 µm) basidiospores, longer pleurocystidia (66–80 × 13.5–16.5 µm), and violaceous tinges in young lamellae and upper parts of the stipe.

## Figures and Tables

**Table 1:** Overview of *Inocybe* isolates used in phylogenetic analyses. Newly generated sequences are in boldface. ^T^ stands for type specimens.

Species	Section	Isolate	Locality	ITS	nrLSU	*rpb2*
*Inocybe aeruginascens*	*Hysterices*	JG270502	Germany	GU949590	JN974970	
*Inocybe aeruginascens*	*Hysterices*	JG310508	Germany	GU949591	MH220256	MH249787
*Inocybe aeruginascens*	*Hysterices*	PC111007	South Africa	GU949592	MH220257	
*Inocybe chondroderma*	*Hysterices*	PBM1760	British Columbia	GU949586	MH220258	
*Inocybe chondroderma*	*Hysterices*	PBM1776	Washington	GU949579	JN974967	MH249789
*Inocybe brijunica* ^T^	*Hysterices*	D. Haelew. F-1610a	Croatia	**MN749370**	**MN749492**	**MT878448**
*Inocybe brijunica* ^T^	*Hysterices*	D. Haelew. F-1610b	Croatia	**MN749371**	**MN749493**	**MT878449**
*Inocybe dulciolens* ^T^	*Lactiferae*	PBM2646	Tennessee	MH216088	MH220265	MH249796
*Inocybe dulciolens*	*Lactiferae*	PBM2450	New York	MH216087	MH220264	MH249795
*Inocybe dulciolens*	*Lactiferae*	LVK13340	New Jersey	MH216084	MH220261	MH249792
*Inocybe erinaceomorpha*	*Lactiferae*	EL128/05	Sweden	AM882735	AM882735	
*Inocybe erinaceomorpha*	*Lactiferae*	JV14756F	Sweden	MH216089	MH220266	MH249797
*Inocybe glabripes*	*Hysterices*	JV7318F	Finland	MH216096		MH249803
*Inocybe hystrix*	*Hysterices*	HRL11842	Quebec	KX897428		
*Inocybe hystrix*	*Hysterices*	PBM3300	North Carolina	GU949588	MH220275	
*Inocybe hystrix*	*Hysterices*	RS31493	Finland		AY380380	AY337381
*Inocybe hystrix*	*Hysterices*	SJ020824	Sweden	AM882810	AM882810	
*Inocybe* aff. *hystrix*	*Hysterices*	REH7405	Costa Rica	GU949589	JN974969	MH249806
*Inocybe melanopus* ^T^	*Hysterices*	Stz3641	Washington		HQ201359	
*Inocybe melanopus*	*Hysterices*	BJ920904	Sweden	AM882725	AM882725	
*Inocybe melanopus*	*Hysterices*	JV4986	Finland	AM882727	AM882727	
*Inocybe melanopus*	*Hysterices*	PBM3975	Tennessee		MH220276	MH249807
*Inocybe melanopus*	*Hysterices*	TAA185135	Estonia	AM882726		
*Inocybe* aff. *pallidobrunnea*	*Hysterices*	PBM1957	Washington	MH216098	MH220277	MH249808
*Inocybe* aff. *pallidobrunnea*	*Hysterices*	PBM2242	Washington	MH216099	JN974968	MH249809
*Inocybe* sp.	*Hysterices*	PBM578	Washington	MH216104	JN974961	MH249813
*Inocybe* sp.	*Hysterices*	TR170-02	New Guinea		JN974964	MH249814
*Inocybe* sp.	*Hysterices*	TR180-02	New Guinea		JN974965	

**Table 2:** Overview of number of characters (total, informative, constant) and selected model of nucleotide substitution, by locus.

Locus	Sequences	Sites	Informative	Constant	Model	-lnL
ITS1	23	246	92	134	HKY + F + G4	1306.690
5.8S	23	158	4	153	TIM3e	249.420
ITS2	23	203	79	113	TPM3u + F + G4	1034.365
nrLSU	25	1379	94	1257	TN + F+I	3068.786
*rpb2*	16	766	156	540	TN + F+I	2935.101

## Supplementary Materials

The following supplementary material is available online at https://www.mdpi.com/2309-608X/7/3/199/s1, File S1: Aligned, concatenated dataset of *Inocybe* sect. *Hysterices*, consisting of five partitions (ITS1, 5.8S, ITS2, nrLSU, *rpb2*).
